# On the Internal Use of Nitrate of Silver in Obstinate Diarrhea and Dysentery

**Published:** 1848-05

**Authors:** Thomas Aikin


*On the Internal Use of Nitrate of Silver in Obstinate Diarrhea and Dysentery.* By THOMAS AIKIN, Esq.—The author of this communication remarks, that the topical application of the nitrate of silver to inflamed or ulcerated mucous surfaces is confessedly a most efficient mode of treating such cases. The knowledge of this fact may have induced physicians to employ the same remedy internally against disease invading the mucous surface of the hollow viscera. Accordingly, we find that ample testimony is afforded to the efficacy of the nitrate of silver in certain morbid conditions of the mucous coat of the stomach; but no English writer, Copland excepted (Dictionary of Medicine,) sanctions its employment as a therapeutic agent in morbid conditions of the mucous surface of the intestinal tube. The author's object in the present communication is to adduce such testimony in favor of its sanative power in these affections as may stimulate further inquiry into the action of this salt in certain obstinate forms of diarrhœa and dysentery, which occasionally resist the action of the most esteemed remedies wielded in the ablest manner.

Boudin (Gazette Méd. No. 51, 1836,) physician to the Military Hospital at Marseilles, treated fifty cases of typhoid fever (dothinenteritis,) in most of which severe diarrhœa was the most prominent feature, with the nitrate of silver thus: When the lower portion of the intestinal tract was presumed to be the seat of ulceration, enemata, containing from one to three grains, dissolved in distilled water, were administered. In most cases one enema sufficed, the symptoms undergoing speedy amelioration. In other cases the remedy was given by the mouth, in half-grain doses every half hour, [?] formed into pills with gum tragacanth, or starch, until from two to four grains were thus taken. In some instances these two modes of treatment were combined; the results were that only two of the fifty cases succumbed. Examination showed "many ulcers" on the mucous membrane in a case of incipient cicatrization—"*en voie de cicatrisation.*" There was evidence of the solution administered per rectum having passed the ileo-cæcal valve, and producing effects on the lower portion of the ileum precisely similar to those resulting from its action on the surface of the colon.

Kalt confirms Boudin's statement, having treated twenty-two cases of dothinenteritis with the nitrate of silver. Of these one died. He gave it in mixture (grs. ij to vj in decoct. salep. oz. vj;) a tablespoonful of which was taken every half hour, or hour, according to circumstances.

Hirsch of Königsberg (Hufeland's Journal) found the nitrate of silver to succeed in obstinate cases of diarrhæa on the failure of ordinary remedies. It proved specially useful in the diarrhea of newly weaned infants. In "the advanced stage of such cases, when emaciation was extreme, the dejections being frequent, fetid, and consisting of a variously colored, sometimes greenish, or bloody mucus, and wanting altogether the fecal character. When apthous ulceration pervaded the mouth, and when prostration was extreme, the action of the nitrate was brilliant." He gave it to children thus:—

℞ Argent nitrat. crystall. gr. ¼.

Aquæ destill. ℥ij,

Gum mimosæ, ℈ij,

Sacch. albi, ʒij. Misce. Ft. mist.

A teaspoonful of this mixture was given every two hours, and an enema, containing a quarter grain of the salt, with mucilage and a little opium, was administered. The good effects of this treatment were occasionally visible in a few hours, sometimes not until the second day. He pronounces it a specific in the diarrhea of infants. He found it almost equally efficacious in severe forms of diarrhea and dysentery occurring in adults. He administered it to the latter in pills, in doses varying from one-twelfth to one-twentieth of a grain every two hours. For this purpose he recommends liquorice powders as preferable to the vegetable extracts which effect its decomposition. He also gave enemata, containing half a grain or a grain, with mucilage and opium.

Canstatt also extols the nitrate of silver as prescribed by Hirsch in the diarrhea ablactatorum.

Since the author became acquainted with Hirsch's observations, opportunity presented for testing the powers of the nitrate of silver in a severe case of diarrhea occurring in a child of a year old. Vomiting and purging set in, and continued with almost unabated intensity for five days. The stomach at length retained fluids in small quantities, but the purging continued. Chalk mixture, kino, opium and acetate of lead were tried, and all, with the exception of the last, seemed to increase the irritation. The dejections were frequent, greenish, sometimes bloody, and very fetid. On the sixth day prostration was very great; there was a tendency to stupor, and quantities of greenish mucus were voided.—Under these circumstances he gave the mixture as prescribed by Hirsch. The first dose seemed to increase the discharges; however, in about six hours, the character of the dejections were improved, they became feculent, and every symptom underwent a corresponding improvement.

Should the foregoing observations induce practitioners in this country to subject the action of the nitrate of silver in diseases of the mucous surface of the intestines to a more extensive tial, they may arrive at results confirmatory of those already obtained by the authorities which the author has quoted, and thereby extend the application of an agent of great therapeutic energy to forms of disease occasionally so intractable as to baffle the powers of ordinary remedies.

[The advantage of the nitrate of silver in the diarrhea of infants, of which we have had considerable experience, is also acknowledged by Bouchart (Manuel Partique des Nou-veaunés) and by Trousseau. We have given it frequently, and with much benefit also, in the "irritable" bowels of the adult. We generally prefer to exhibit it in solution, more especially in children, since, if given in pill or powder, we have no guarantee that it will not, by suddenly dissolving, exert all its effects, which, in that case, may be too powerful, upon a circumscribed portion of the mucous membrane. This is a point which is not sufficiently attended to in prescribing the nitrate of silver for gastrodynia, and sufficiently accounts for the diversity of opinion respecting its benefit in this complaint. It may be readily conceived that it makes all the difference whether half a grain of solid nitrate of silver lies in a corner of the stomach and dissolves, or whether originally in solution its action is distributed throughout the entire irritable mucous membrane.]—*Ibid.*

